# Acute and Chronic Effect of Physiological Factors on Arterial Stiffness

**DOI:** 10.3390/jcm12083044

**Published:** 2023-04-21

**Authors:** Shigehiko Ogoh

**Affiliations:** 1 Department of Biomedical Engineering, Toyo University, Kawagoe 350-8585, Japan; ogoh@toyo.jp; 2 Neurovascular Research Laboratory, University of South Wales, Pontypridd CF37 4AT, UK

Arterial stiffness is a disease of the arterial media, and it is well known that it is accelerated by aging. Arterial stiffness, vascular biomarkers such as central hemodynamics, wave reflections, and endothelial function, is associated with cardiovascular diseases (CVD) such as hypertension, diabetes, and dyslipidemia due to changes in the mechanical properties of the arterial walls. A recent epidemiological study [1] investigated the biological determinants of arterial stiffness (arterial stiffness index (ASI) > 10 m/s) among healthy participants, and, as a result, ASI was associated with multiple parameters (age, BMI, cystatin c, phosphate, mean arterial pressure, etc.). Interestingly, the decline in systolic blood pressure (SBP) associated with repeated cuff-oscillometric inflation [2] or heart rate variability [3] was significantly correlated with the ASI. Since CVD is associated with changes in repeated measuring of SBP or impaired cardiac autonomic function, these recent findings indicate that arterial stiffness, or atherosclerosis, is a subclinical marker associated with CVD. Thus, it is strongly recommended to identify the markers of increased arterial stiffness to support the management of future CVD risks through preventive strategies.

Recently, based on this concept, Anastasio et al. [4], in the Journal of Clinical Medicine, demonstrated that the non-invasive measurements of arterial stiffness were strong prognostic parameters in patients with heart failure who had been discharged after an acute heart failure decompensation. In this study, the authors evaluated the prognostic role of arterial stiffness in patients with heart failure who had been discharged after an acute episode of decompensation. They accomplished this by evaluating cut-off values for clinical assessment and demonstrated that arterial stiffness is inversely correlated with free-event survival. In addition, Gagliardino et al. [5] evaluated arterial stiffness indicators in people with prediabetes and its possible pathogenesis. They demonstrated that the arterial stiffness index was significantly impaired in people with metabolic syndrome. These findings indicate that screening for arterial stiffness could be useful for reducing the mortality and morbidity associated with CVD, as well as the management and primary and secondary prevention of CVD events [6].

It has been established that arterial stiffness is determined by chronic physiological factors: age; blood pressure; and unhealthy lifestyles such as an inappropriate diet, a lack of physical activity, and smoking-induced reduction in the elasticity of the arteries due to a structural change of the arterial wall [7]. However, arterial stiffness may be altered acutely via functional change. For example, orthostatic stress increases arterial stiffness acutely [8]. This finding indicates the possibility that this attenuated arterial stiffness is caused by a functional change rather than a structural change in the artery, developed due to lifestyle-induced modification. Thus, to evaluate arterial stiffness associated with CVD, it is important to consider both acute and chronic effects of arterial stiffness and to distinguish between them.

Chronic exercise is effective in reducing arterial stiffness in the long term. On the other hand, an acute effect of exercise on the arterial stiffness index has been observed, although it returned to the baseline level in the following 24 h [9]. This finding suggests that the acute effect of exercise on arterial stiffness is probably a functional rather than a structural effect. Thus, it remains unclear that repeated acute exercise-induced functional change could result in a chronic structural effect; in other words, it is unknown how the repeated acute effect of exercise improves arteriosclerosis. Another interesting study [10] examined the effect of objectively measured physical activity on arterial stiffness in children and adolescents with congenital heart disease. However, the authors did not find an effect of physical activity on arterial stiffness in children with cardiovascular disease, and they concluded that these results may have been due to low activity or short exposure to the respective risk factors, as the patients were children. Thus, the acute effect of exercise may not predict the effect of chronic exercise on arterial stiffness. In other words, chronic exercise-induced improvements in arterial stiffness do not result from repeated acute exercise effects. Regarding the acute effect on arterial stiffness, it has been reported that caffeine increases arterial stiffness and also has an impact on the cardiovascular system [11]. In addition, interestingly, an acute elevation of arterial stiffness has been observed in a cohort of healthy children and teenagers after energy drink consumption [12]. This finding suggests that those with pre-existing health conditions such as arterial hypertension, diabetes, overweight, or congenital heart disease, should particularly be discouraged from energy drink consumption.

With this background, we need to point out two matters. First, the measurement of arterial stiffness should be an important indicator of the index of risk of cardiovascular disease (CVD); however, the difference between acute and chronic effects or between functional and structural effects should be distinguished in order to assess arterial stiffness. In the other words, it may be considered that chronic effects on arterial stiffness need to be isolated from acute effects if CVD risk is evaluated. Second, many researchers have investigated the effect of acute interventions, such as exercise, on arterial stiffness based on the concept that a repeated acute effect causes a chronic effect. However, the acute effect on arterial stiffness is completely different from the chronic effect; the acute effect may be caused by functional modification, but in contrast, the chronic effect may be caused by a structural effect. Thus, it remains unclear whether repeated acute changes in arterial stiffness have chronic effects.
